# Tolerance of High-Intensity Focused Ultrasound Ablation in Patients with Hepatocellular Carcinoma

**DOI:** 10.1007/s00268-012-1660-7

**Published:** 2012-06-15

**Authors:** Tan To Cheung, Ferdinand S. K. Chu, Caroline R. Jenkins, Dickson S. F. Tsang, Kenneth S. H. Chok, Albert C. Y. Chan, Thomas C. C. Yau, See Ching Chan, Ronnie T. P. Poon, Chung Mau Lo, Sheung Tat Fan

**Affiliations:** 1 Department of Surgery, The University of Hong Kong, 102 Pokfulam Road, Hong Kong, China; 2 Department of Radiology, Queen Mary Hospital, Hong Kong, China; 3 Department of Anaesthesiology, The University of Hong Kong, Hong Kong, China; 4 State Key Laboratory for Liver Research, The University of Hong Kong, Hong Kong, China

## Abstract

**Background:**

High-intensity focused ultrasound (HIFU) ablation is a relatively new, noninvasive way of ablation for treating hepatocellular carcinoma (HCC). Emerging evidence has shown that it is effective for the treatment of HCC, even in patients with poor liver function. There is currently no data on the safety limit of HIFU ablation in patients with cirrhosis. However, this information is vital for the selection of appropriate patients for the procedure. We analyzed HCC patients who had undergone HIFU ablation and determined the lower limit of liver function and other patient factors with which HCC patients can tolerate this treatment modality.

**Methods:**

Preoperative variables of 100 patients who underwent HIFU ablation for HCC were analyzed to identify the risk factors in HIFU intolerance in terms of stress-induced complications. Factors that may contribute to postablation complications were compared.

**Results:**

Thirteen (13 %) patients developed a total of 18 complications. Morbidity was mainly due to skin and subcutaneous tissue injuries (*n* = 9). Five patients had first-degree skin burn, one had second-degree skin burn, and three had third-degree skin burn. Four complications were grade 3a in the Clavien classification and 14 were below this grade. Univariate analysis showed that age (*p* = 0.022) was the only independent factor in HIFU intolerance.

**Conclusions:**

HIFU ablation is generally well tolerated in HCC patients with cirrhosis. It is safe for Child-Pugh A and B patients and selected Child-Pugh C patients. With this new modality, HCC patients who were deemed unsalvageable by other surgical means in the past because of simultaneous Child-Pugh B or C disease now have a new hope.

## Introduction

Advances in surgical techniques have improved the survival of patients with hepatocellular carcinoma (HCC) considerably during the past years. However, as few as 25 % of HCC patients are amenable to surgical resection on presentation. The low resectability rate is mainly due to marginal liver function reserve, a condition secondary to chronic liver disease, such as cirrhosis [1]. Locoregional ablative therapies offer good alternatives to resection for HCC patients who are awaiting liver transplantation, a treatment modality that benefits far fewer patients than it should because of the severe shortage of liver grafts [2, 3].

Among various methods of ablation for HCC, radiofrequency ablation (RFA) is relatively popular because it is effective, simple, and repeatable [4, 5]. Nonetheless, it is an invasive procedure and, according to a review of reports involving 3,670 patients, it had an overall complication rate of 8.9 % [6, 7]. RFA involves direct tumor puncturing, which may precipitate direct tumor seeding, particularly when the lesion is close to a major hepatic vessel. For some patients, such as those with gross ascites, RFA is not viable at all. High-intensity focused ultrasound (HIFU) ablation is a newly emerged alternative that seems promising for the management of HCC, even for patients with advanced cirrhosis and gross ascites [8].

### HIFU modality

HIFU treatment is a totally extracorporeal noninvasive ablation mode using focused ultrasound energy that is capable of causing coagulative necrosis of the targeted HCC via intact skin without the need of surgical incision [9, 10]. It utilizes a unique frequency of ultrasound wave of 0.8–3.5 MHz, which can be focused at a distance from the therapeutic transducer. The accumulated energy at the focused region induces necrosis of the target lesion by elevating the tissue temperature to greater than 60 °C. Temperature outside the focus point remains static as particle oscillation remains minimal. Because ultrasound energy travels much better in water than in air, the presence of ascites in HCC patients actually facilitates energy propagation to the targeted HCC. Passage of energy without the puncture of a physical instrument and its superior performance in patients with ascites give HIFU treatment superiority over other treatment modalities for HCC.

Initial results of HIFU ablation for the management of HCC were promising with a complete ablation rate of 28.5–68 % for single treatment [8, 11, 12]. Once considered a “chinoiserie,” HIFU ablation is gaining popularity in other parts of the world [13–15]. But unlike partial hepatectomy or RFA for HCC patients with cirrhosis, there are limited data on the safety limits for HIFU ablation, yet this kind of information is vital for the selection of appropriate patients for the procedure. To our knowledge, no study has evaluated HCC patients’ tolerance of HIFU ablation. In this study, we analyzed the results of HIFU ablation in HCC patients and determined the lower limit of liver function with which HCC patients can tolerate this treatment modality.

## Patients and methods

From October 2008 to June 2010, 100 patients received HIFU ablation for HCC at our center. They all underwent a complete set of liver function tests. Diagnosis of HCC was based on two typical imaging findings, together with cytology and histopathology examination results if previous surgery had been performed. Contrast magnetic resonance imaging scans were interpreted by consultant radiologists. A lesion was considered HCC-positive if it demonstrated the typical vascular pattern of HCC (i.e., hypervascular in the arterial phase with washout in the portal venous phase) and an increased signal of lesion in T2W image. A serum alpha-fetoprotein level >400 ng/ml was considered significantly diagnostic of HCC.

Upon diagnosis of HCC, patients were evaluated for treatment according to the following general principles. Surgical resection is offered to patients with suitable tumors and liver function that can tolerate it as decided by indocyanine green retention test and liver volumetry. Open RFA is offered for tumors <5 cm if surgical resection is deemed risky. Liver transplantation is considered for patients with poor liver function and tumors within the UCSF criteria. Percutaneous RFA by interventional radiologist is offered to patients who have unresectable HCC due to poor liver function and patients who refuse open RFA and surgical resection. HIFU ablation is offered to patients not suitable for percutaneous RFA but with satisfactory general condition as assessed by anesthesiologist. Transarterial chemoembolization (TACE) is used to treat nonablatable tumors. Patients with any of the following problems are precluded from HIFU therapy: tumor invasion of a major hepatic blood vessel; extrahepatic metastasis of disease; unsuitability for general anesthesia; receiving medical treatment that may cause skin complication (e.g., targeted therapy).

**Fig. 1:**
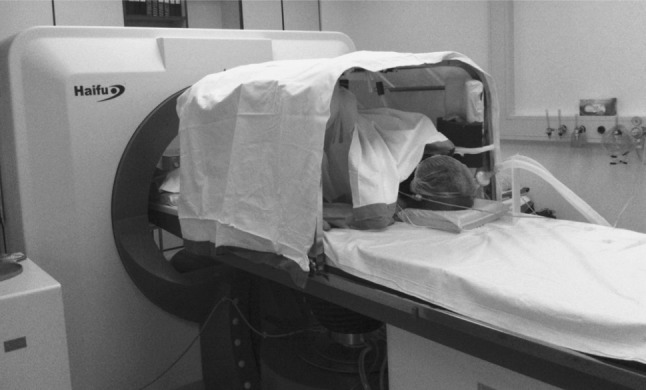
After general anesthesia, patient on the platform was put in the right lateral position. The transducer below the platform targeted the liver lesion through the rib spaces

HIFU ablation is an accepted and standard practice of treatment for unresectable HCC at our center [16]. All HIFU treatments in the present series were performed by our center’s HIFU team consisting of experienced surgeons in hepatobiliary and pancreatic surgery and experienced radiologists. The first author attended every HIFU treatment since 2007, involving 80 % of all cases performed. Our center has been employing the JC HIFU system (Chongqing Haifu Technology, Chongqing, China) for HIFU treatment. The JC HIFU system comprises a real-time diagnostic imaging unit, a therapeutic unit, a degassed water circulation unit, and a computer system. The real-time diagnostic imaging unit provides direct visualization of the tumor. The therapeutic unit consists of an ultrasound energy transducer which focuses the ultrasound energy at a 12-cm focal point. The degassed water circulation unit provides a medium for ultrasound transmission outside the body. The computer system controls these three units. The actual operative setting is shown in Fig. 1.

All patients were subjected to general anesthesia to aid their comfort, because the whole procedure could last for 3 h and the patients had to lie still and were subjected to long periods of breath-holding. In addition, general anesthesia allowed manipulation of tumor location by inducing the Valsalva maneuver during the procedure by the anesthesiologist. Artificial right pleural effusion before treatment was required for the ablation of lesions adjacent to the ribs or close to the dome of the liver to displace the lung parenchyma from the pathway of the ultrasound beam and to provide an acoustic window for the treatment. After anesthetic induction, the patient was placed in a left-lateral position and pleural puncture was performed at the right fourth intercostal space along the midaxillary line, using an 18-gauge Tuohy needle with a passive loss of resistance to a falling column of fluid. Approximately 800 ml of warm normal saline was required. Ventilation was suspended in the expiratory phase with an open adjustable pressure limit valve when the needle was introduced to the pleural cavity. HIFU treatment was performed under real-time ultrasound image guidance. Target lesions were localized by a 3.6-MHz diagnostic ultrasound probe (Philips) incorporated at the center of the transducer. Diagnostic and therapeutic ultrasound waves were emitted synchronously in the same direction. Detailed planning was performed according to the tumor size and location as shown on the computer monitoring module, which was synchronized with the real-time diagnostic imaging probe. Parallel slices of the target tumor with 5-mm separations were planned and then ablated slice by slice with focused ultrasound energy produced by the transducer operating at 0.8 MHz. Grey-scale changes of the ablated sites were observed during the ablation procedure, indicating the temperature change inside the target lesion [17].

Postoperative blood tests for complete blood picture, prothrombin time and liver and kidney functions were performed routinely on days 1, 3, 7, and 14, or according to specific clinical situations. All patients were closely observed for any development of complications and had routine blood tests according to the protocol. Contrast computed tomography or magnetic resonance imaging was performed 1 month after treatment. The results were interpreted by a consultant radiologist together with another radiologist. Complete ablation was defined as full coverage of the lesion by HIFU scar at the first imaging. Repeated HIFU was performed for incomplete ablation. Reasons for readmission were recorded. Cases in which complications developed after HIFU treatment were analyzed to determine the risk factors for intolerance of the treatment. Intolerance was defined as one or more complications occurring secondary to the procedure.

For this study, intractable ascites was defined as a collection of abdominal fluid resistant to diuretics or a collection of abdominal fluid requiring paracentesis. “Massive” pleural effusion was that which required drainage for dyspnea or desaturation. Encephalopathy was defined as development of impairment of the conscious state, the diagnosis of which was supported by blood ammonia levels. Hyperbilirubinemia >100 μmol/l was considered an indicator of liver failure. Hyperkalemia was present when serum potassium level was >6.0 mmol/l, requiring medical treatment for correction.

### Statistical analysis

The Mann–Whitney *U* test was used to compare continuous variables, and a chi-squared test was used to compare discrete variables. Univariate analysis was used to identify risk factors for patients developing serious complications. Parameters included patient age, preprocedure levels of serum bilirubin, albumin, alanine transaminase and aspartate transaminase, platelet count, international normalized ratio, total diameter of HCC, ablation time, ablation energy, presence of cirrhosis on imaging, and comorbid illness. Statistical significance was defined as *p* < 0.05. All statistical calculations were performed by using the SPSS/PC + computer software (SPSS, Chicago, IL).

## Results

The 100 patients included in the study comprised 78 males and 22 females, aged 34 to 85 (median, 65) years. Their preoperative characteristics can be viewed in Table 1. Premorbid medical diseases were present in 69 patients. These diseases included hypertension (*n* = 27), ischemic heart disease (*n* = 9), diabetes mellitus (*n* = 15), renal impairment (*n* = 4), and other comorbidities (*n* = 20). Twenty-seven patients received HIFU ablation as a primary treatment of HCC, and three patients received it as a bridging therapy before liver transplantation. It also was delivered as treatment of recurrence of HCC after TACE (*n* = 41), partial hepatectomy (*n* = 28), and liver transplantation (*n* = 1).

**Table 1:** Preoperative characteristics of the 100 patients

	No. of patients (unless otherwise stated)
Median age, year (range)	65 (34–85)
Male:female ratio	78:22
Median tumor size on CT scan, cm (range)	2.2 (0.9–8.0)
Median number of tumors to be treated (range)	1 (1–2)
Hepatitis B virus carrier	80
Hepatitis C virus carrier	15
Presence of ascites	15
Cirrhotic liver on imaging	34
Child-Pugh grade A	84
Child-Pugh grade B	15
Child-Pugh grade C	1
ASA class 1	1
ASA class 2	65
ASA class 3	34
MELD score > 14	5

*ASA* American Society of Anesthesiologists, *MELD* model of end-stage liver disease

Thirteen (13 %) patients developed a total of 18 complications, either intraoperative or postoperative. Four complications were grade 3a in the Clavien classification and 14 were below this grade (Table 2). Univariate analysis was performed to evaluate the 20 factors that might influence the patients’ tolerance of treatment (Table 3), among which 1 factor was found to be significant and 19 factors were found to be insignificant. The only significant factor was patient age (63 vs. 72 years, *p* = 0.022). The complete ablation rate with single treatment was 87 % for tumors <3 cm. No hospital mortality occurred during the study period.

**Table 2:** Intraoperative and postoperative complications developed in the 13 patients

Complication	Number
Skin burn	
First degree	5
Second degree	1
Third degree	3
Pleural effusion	1
Chest infection	1
Renal impairment	1
Variceal bleeding	1
Right chest wall bruising	1
Hyperbilirubinemia	1
Liver abscess	1
Pneumothorax	2
Total no. of complications	**18**
Clavien classification	
3a	4
3b	0
4	0
5	0

Bold value indicates statistical significance (*p* < 0.05)

**Table 3:** Factors that might influence the patients’ tolerance of HIFU

	No complication (*n* = 87)	With complication (*n* = 13)	*p* value
Age (year)	63 (34–85)	72 (51–75)	**0.022**
Male:female ratio	68:19	10:3	1
ASA classification			0.804
1	1 (1.3 %)	0	
2	52 (65.8 %)	13 (61.9 %)	
3	26 (32.9 %)	8 (38.1 %)	
Child-Pugh grade			0.927
A	73	11	
B	13	2	
C	1	0	
Hepatitis B	70	10	1
Hepatitis C	12	3	0.647
Ascites	14	1	0.679
Cirrhotic liver on imaging	30/87 (34.48 %)	4/13 (30.76 %)	0.72
Alpha-fetoprotein (ng/ml)	18 (2–156600)	15 (2–1516)	0.538
Bilirubin (μmol/l)	14 (4–46)	14 (7–37)	0.762
Albumin (g/dl)	39 (24–50)	37 (27–42)	0.290
Aspartate transaminase (μ/l)	47 (18–308)	40 (21–78)	0.198
Platelet count (×10^9^/l)	99 (26–248)	121 (61–268)	0.23
International normalized ratio	1 (0.8–1.5)	1.1 (1–1.4)	0.601
Tumor size on CT scan (cm)	2.2 (0.9–8.0)	2.1 (1–5.7)	0.063
No. of tumors treated	1 (1–2)	1 (1–2)	0.295
Location of tumor			0.204
Section 2	1 (1.2 %)	2 (15.4 %)	
Section 3	5 (6.0 %)	1 (7.7 %)	
Section 4	5 (6.0 %)	0 (0 %)	
Section 5	4 (4.8 %)	1 (7.7 %)	
Section 6	10 (11.9 %)	2 (15.4 %)	
Section 7	19 (22.6 %)	1 (2.8 %)	
Section 8	22 (26.2 %)	3 (23.1 %)	
Multiple ablations	21 (24.2 %)	3 (23.1 %)	
Energy (J)	559906 (34477–19411678)	632641 (124414–1509327)	0.726
Average HIFU acoustic power (W)	370 (155–473)	380 (120–450)	0.806
HIFU exposure time (s)	1427 (135–7487)	1823 (338–5888)	0.575

Bold value indicates statistical significance (*p* < 0.05)

## Discussion

Management of HCC remains a great challenge to clinicians despite new treatment modalities that keep emerging and surgical techniques that keep improving. The challenge is due to the fact that HCC is frequently accompanied by cirrhosis. The combination of these two detrimental conditions leads to an overall poor outcome in HCC patients. Only 25 % of patients can be subjected to surgical intervention upon diagnosis [18–20].

Many centers, including those with little experience in resection of liver tumors for patients with cirrhosis, are using RFA as a treatment modality due to its simplicity, especially when compared with hepatectomy. However, RFA is considered relatively contraindicated in patients with advanced cirrhosis or gross ascites. In these patients, the complication rate remains high.

**Fig. 2:**
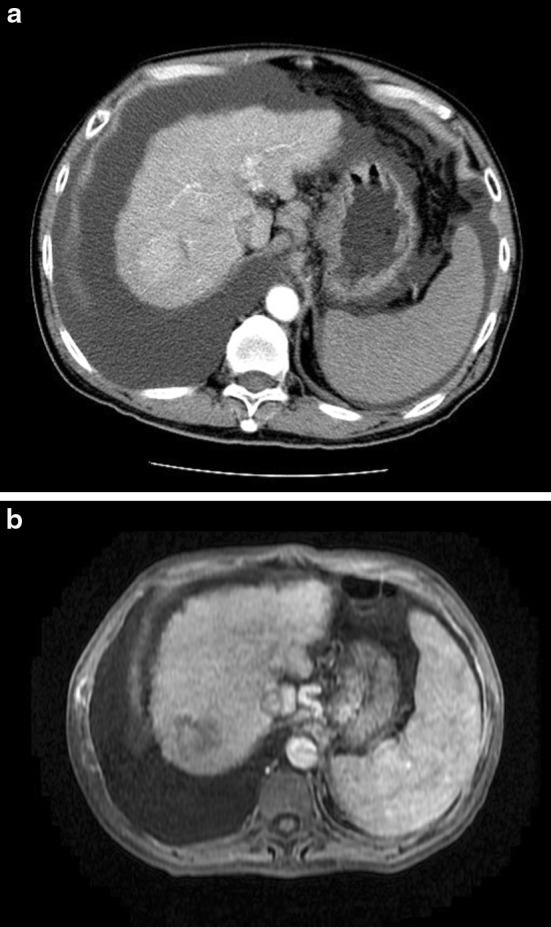
**a** Computed tomography scan showing a hepatocellular carcinoma at the dome of liver. The liver is of Child-Pugh C cirrhosis with gross ascites. **b** Magnetic resonance imaging scan of the same patient 1 month after high-intensity focused ultrasound (HIFU) ablation. Complete ablation is noted in this scan. The patient’s liver functions did not deteriorate from their pre-HIFU status

For HIFU treatment, effective propagation of ultrasound energy depends on a conductive medium with high density. Thus, it can be performed safely in patients with gross ascites, as shown in Fig. 2a, b. The ascites inside the peritoneal cavity not only provides a clear image for the diagnostic ultrasound probe but also serves as a good medium for energy transfer. The presence of ascites also protects subcutaneous tissue from being damaged by the focused ultrasound energy. So, poor liver function with ascites is no longer a contraindication to HCC treatment.

In the present series, all HIFU ablations were performed by a single team of experienced hepatic surgeons and interventional radiologists to prevent bias that might otherwise be brought about by operator variations. The morbidity rate in the study was 13 %, which is comparable to the morbidity rate after RFA in the same center. In fact, many recent studies reported higher complication rates of RFA, ranging from 13 to 20 % [21–23].

Although the ultrasound energy in HIFU treatment is focused at the lesion, skin and subcutaneous tissue burns did occur [8, 24]. Thermal injury can occur adjacent to the target lesion or along the ultrasound beam pathway, particularly when treating subcapsular HCC that is located closely to the subcutaneous area. Ultrasound possesses the properties of sound waves, i.e., reflection, absorption, refraction, and the cavitation effect of energy. These unique features of ultrasound energy impart its diagnostic power by allowing visualization of the internal organ, expression of the lesion by different ultrasound absorption characteristics, and visualization of tumor destruction during treatment. When the power of ultrasound is increased in the treatment mode, some of the energy is absorbed along the targeted lesion pathway. Most skin burns occurred with tumors located near the subcapsular area, where some of the energy was reflected by the ribs overlying the tumor, and the abdominal wall was the first tissue to absorb the energy.

There are two postulated mechanisms through which HIFU damages the subcutaneous area: reflection from the gas cavity and reflection by the ribs. Dubinsky et al. [25] suggested that the reflection of ultrasound beam by the lung parenchyma and bowel gas could cause burning of the abdominal wall. The multiple layers of the abdominal wall could be a good substance for absorption of energy present in the gas-filled cavity inside the body. This is observed more frequently in elderly patients, who are more likely to have chronic obstructive airway diseases than younger patients. A rib along the ultrasound beam also could absorb excessive energy [26]. Although HIFU has an accurate focusing property, the penetration of ultrasound wave is largely hindered by the presence of ribs, particularly when the tumor is close to the dome or the posterior section of the liver, where the spaces between ribs are narrower. In addition, bones have a high ultrasound absorption coefficient. This can cause overheating of the overlying subcutaneous tissue while effective energy may not be transferred to the targeted area.

To overcome the problem of reflection of HIFU, artificial pleural effusion is required for the ablation of lesions adjacent to the ribs or close to the dome of the liver. This is to displace the lung parenchyma from the pathway of the ultrasound beam and to provide an acoustic window for the treatment. Two patients in the present series developed pneumothorax during introduction of hydrothorax. Although breathing is suspended, the introduction of needle is a blind procedure and the depth of the penetration varies from patient to patient as the subcutaneous tissue’s thickness cannot be accurately predicted.

Theoretically, the introduction of hydrothorax can lead to lung collapse and intrapulmonary shunting can occur. Nonetheless, only one patient in the present series developed intolerance and required drainage of pleural effusion. Another patient, a chronic smoker, developed a chest infection after the procedure, which fully resolved after vigorous chest physiotherapy and antibiotics. In the majority of patients, the artificial pleural effusion was rapidly absorbed after the procedure as shown by postoperative serial chest X-ray investigations. Miserocchi [27] reported that introduction of artificial pleural effusion actually increases lymphatic drainage of the chest wall cavity.

Ventilation control can be a stressful challenge to patients receiving HIFU treatment, especially for elderly patients and patients with predisposing pulmonary disease. To execute precise ablation as planned, immobilization is required for lesions that move with respiration. Intermittent breath holding at sustained airway pressure is necessary during the procedure. A period of high positive inspired pressure for up to 40 s can affect the cerebral blood flow, which is already reduced in the elderly [28]. Fortunately, no encephalopathy or cerebral vascular accident was noted in the present series.

In the present study, patient age was the only significant factor for treatment intolerance, as with other operations that require general anesthesia. Aging is an inevitable physiological phenomenon characterized by degenerative changes in both the structure and the functional capacity of organs. Elderly patients are prone to both dehydration and fluid overload because of their weakened ability to concentrate and dilute urine and to handle sodium. Chronic obstructive pulmonary disease and pneumonia are very common among the elderly. Closing volume increases with age, and forced expiratory volume in 1 s declines 8–10 % per decade due to reduced pulmonary compliance. As a result, risks of pneumothorax, pneumonia, and pleural effusion increase when the Valsalva maneuver is applied during general anesthesia.

Although the median size of tumors was 2 cm in the present series, an 8-cm tumor in a patient with Child-Pugh B cirrhosis was ablated with HIFU. This patient was denied hepatectomy and RFA because of his poor liver function and big tumor size. He tolerated the HIFU treatment well and did not develop any complication. Unlike other thermal ablative therapies, such as RFA and microwave ablation, which produce heat that inevitably damages the surrounding structure, HIFU produces very little collateral damage beyond the targeted lesion. This is due to the unique feature of its energy dissipation process. In HIFU treatment, energy is transferred to the lesion at a very slow pace. Each cycle of energy emission is separated by 1–2 min of resting, and each energy emission creates an area of coagulative necrosis 1 cm only in diameter. The surrounding liver parenchyma receives very little energy and thus hardly gets any damage. This was evidenced by the goods results of the present series as shown by postoperative liver function tests and 1-month imaging scans. No ablation of any unplanned area was found on the scans.

Very poor liver function, such as that possessed by patients with Child-Pugh B or C cirrhosis, often gives rise to more complications after therapies, such as TACE, RFA, and hepatectomy. It seems, however, that HIFU can deal with very poor liver function successfully. Among 100 patients, there were 84 Child-Pugh A, 15 Child-Pugh B, and 1 Child-Pugh C, yet no association between intolerance of treatment and liver function was found. It is believed that HIFU is safe for patients with gross ascites, which is a sign of decompensated cirrhosis.

## Conclusions

This study is the first to examine HCC patients’ tolerance of HIFU and to analyze factors that affect such tolerance. Patient age is the only factor that was found to be significant in HIFU intolerance. HIFU ablation is a generally well-tolerated treatment modality. It is safe for Child-Pugh A and B patients and selected Child-Pugh C patients. With this new modality, HCC patients who were deemed unsalvageable by other surgical means in the past because of simultaneous Child-Pugh B or C disease now have a new hope.
